# Emerging role of antidiabetic drugs in cardiorenal protection

**DOI:** 10.3389/fphar.2024.1349069

**Published:** 2024-02-06

**Authors:** Wen-Jia Fu, Jin-Ling Huo, Zi-Hui Mao, Shao-Kang Pan, Dong-Wei Liu, Zhang-Suo Liu, Peng Wu, Zhong-Xiuzi Gao

**Affiliations:** ^1^ Traditional Chinese Medicine Integrated Department of Nephrology, The First Affiliated Hospital of Zhengzhou University, Zhengzhou, China; ^2^ Institute of Nephrology, Zhengzhou University, Zhengzhou, China; ^3^ Henan Province Research Center for Kidney Disease, Zhengzhou, China; ^4^ Key Laboratory of Precision Diagnosis and Treatment for Chronic Kidney Disease in Henan Province, Zhengzhou, China

**Keywords:** diabetes mellitus, cardiorenal protection, SGLT2 inhibitors, GLP-1 receptor agonists, DPP-4 inhibitors

## Abstract

The global prevalence of diabetes mellitus (DM) has led to widespread multi-system damage, especially in cardiovascular and renal functions, heightening morbidity and mortality. Emerging antidiabetic drugs sodium-glucose cotransporter 2 inhibitors (SGLT2i), glucagon-like peptide-1 receptor agonists (GLP-1RAs), and dipeptidyl peptidase-4 inhibitors (DPP-4i) have demonstrated efficacy in preserving cardiac and renal function, both in type 2 diabetic and non-diabetic individuals. To understand the exact impact of these drugs on cardiorenal protection and underlying mechanisms, we conducted a comprehensive review of recent large-scale clinical trials and basic research focusing on SGLT2i, GLP-1RAs, and DPP-4i. Accumulating evidence highlights the diverse mechanisms including glucose-dependent and independent pathways, and revealing their potential cardiorenal protection in diabetic and non-diabetic cardiorenal disease. This review provides critical insights into the cardiorenal protective effects of SGLT2i, GLP-1RAs, and DPP-4i and underscores the importance of these medications in mitigating the progression of cardiovascular and renal complications, and their broader clinical implications beyond glycemic management.

## 1 Introduction

Diabetes mellitus (DM) is the most common metabolic disorder worldwide. It is reported that the prevalence of diabetes will increase to 12.2% (789.2 million) by 2045 (Sun et al., 2022). Type 2 diabetes mellitus (T2DM) is particularly prone to a range of complications, including macrovascular disease (cardiovascular and cerebrovascular disease), which is mainly characterized by atherosclerosis of large blood vessels, as well as microvascular disease (diabetic retinopathy and diabetic kidney disease), which often manifests as microvascular endothelial dysfunction and microthrombosis (Li et al., 2023; Mauricio et al., 2023). The driving factors for cardiovascular complications in diabetic patients including glucotoxicity, lipotoxicity, and hypertension (Kenny and Abel, 2019). Meanwhile, diabetic kidney disease (DKD) is the most common cause of death among microvascular complications of diabetes and is closely associated with cardiovascular outcomes (Blazek and Bakris, 2023). Currently, there has been a paradigm shift in the management of diabetes and its complications, with a focus on not only controlling blood glucose levels but also addressing the associated cardiovascular and renal risks.

In recent years, new classes of anti-diabetic medications such as sodium-glucose cotransporter 2 inhibitors (SGLT2i), glucagon-like peptide-1 receptor agonists (GLP-1RAs), and dipeptidyl peptidase-4 inhibitors (DPP-4i), have shown efficacy in reducing cardiovascular events, slowing the progression of DKD, and improving overall cardiovascular and renal health in diabetic patients (Yin et al., 2022; Guo et al., 2023; Klen and Dolžan, 2023; Panico et al., 2023). More importantly, these medications exhibit cardiorenal protective effects beyond glycemic control and have the potential to ameliorate non-diabetic cardiovascular and renal diseases.

Research have shown that these three classes of drugs can reduce oxidative stress and inflammation through various mechanisms, including reducing cell damage caused by advanced glycation end products, improving mitochondrial function, and inhibiting the production of reactive oxygen species. This suggests that these medications may have therapeutic potential beyond lowering glucose levels (Andreadi et al., 2023; Balogh et al., 2023). Understanding the mechanisms of these drugs is crucial for developing targeted therapies and improving the quality of life for millions of individuals affected by diabetes-related complications.

## 2 Emerging antidiabetic drugs

### 2.1 SGLT2i

Sodium-glucose cotransporter 2 (SGLT2) is located in the proximal tubules of the kidney and is responsible for reabsorbing 80%–90% of urine glucose. Studies have shown that SGLT2 expression is upregulated in the tubular tissues of T2DM and type 1 diabetes mellitus (T1DM) patients (Rahmoune et al., 2005). SGLT2i reduce glucose reabsorption by inhibiting this protein. Interestingly, this effect is independent of insulin secretion and β-cell function, largely reducing the burden on β-cells and the risk of hypoglycemia (Abdul-Ghani et al., 2013). The reduced glucose reabsorption also results in less fluid retention and better control of overweight and hypertension, which often accompany T2DM. Over the past decade, SGLT2i have become a hot topic in scientific and clinical research and a breakthrough in the field of new hypoglycemic agents because of their unique therapeutic effect on diabetes. Representative drugs include empagliflozin, canagliflozin, dapagliflozin, sotagliflozin, ertugliflozin, etc.

### 2.2 GLP-1RAs

Glucagon-like peptide-1 (GLP-1) is an incretin hormone that is secreted in large amounts by L cells located in the intestinal crypt when the intestine is stimulated by nutrients (Müller et al., 2019). The action of GLP-1 depends on the location of its receptors. GLP-1 receptors belong to the G protein-coupled receptors family and are widely distributed in various tissues of the body. In pancreatic α cells, GLP-1 can reduce the secretion of glucagon, while in β cells, it can increase the secretion of insulin, improve the body’s insulin sensitivity, and even promote the proliferation of β cells (Graaf et al., 2016). What’s more, GLP-1, which is located in the brain, suppresses appetite and reduces food intake, leading to weight loss, which is as important as glycemic control in patients with T2DM (Baggio and Drucker, 2014). GLP-1RAs are a new class of antidiabetic drugs that were first approved for the treatment of diabetes in 2005. The representative drugs are exenatide, dulaglutide, liraglutide, and semaglutide.

### 2.3 DPP-4i

Dipeptidyl peptidase-4 (DPP-4) is an enzyme that can rapidly cleave GLP-1, which is a hormone with a very short half-life (Tschöp et al., 2023). DPP-4i can effectively prolong the half-life of GLP-1, increase insulin in the body, and reduce blood glucose over a long period (Capuano et al., 2013). The representative drugs are sitagliptin, linagliptin, and saxagliptin.

## 3 Cardiorenal protection of SGLT2i, GLP-1RAs, and DPP-4i

The American Diabetes Association (ADA) recommends that SGLT2i and GLP-1RAs be used in combination with metformin as first-line initial therapy in patients at high risk of heart failure (HF), atherosclerotic cardiovascular disease (ASCVD), and chronic kidney disease (CKD). In patients with T2DM, GLP-1RAs are even more effective than insulin in some cases. For patients with established ASCVD, SGLT2i and GLP-1RAs can be used as additional agents alone, independent of metformin (Committee ADAPP, 2022b). Compared with the former two agents, DPP-4i are slightly inferior, and studies on cardiac and renal outcomes are limited. DPP-4i can be considered in patients with GLP-1RAs intolerance. Furthermore, the ADA also states that SGLT2i should be administered as early as possible in patients with stage CKD2 or worse, regardless of blood glucose. GLP-1RAs are principally used to delay cardiovascular disease, which may also delay CKD progression (Committee ADAPP, 2022a).

Similarly, the 2022 Kidney Disease: Improving Global Outcomes (KDIGO) guidelines also recommend SGLT2i therapy in patients with T2DM and CKD. Long-acting GLP-1RAs are recommended when ideal glycemic targets are not achieved with the combination of metformin and SGLT2i. Notably, when GLP-1RAs combined with insulin or sulfonylureas, reduced dose of these drugs is recommended to avoid hypoglycemia. Some DPP-4i, such as saxagliptin and sitagliptin, are accessible to patients with an estimated glomerular filtration rate (eGFR) of less than 30 mL/min/1.73 m^2^ or who are receiving dialysis, and offer a viable alternative for individuals who are not utilizing GLP-1RAs (de Boer et al., 2022).

In general, SGLT2i and GLP-1RAs have significant beneficial effects on renal and cardiac outcomes, while the cardiorenal protective effect of DPP-4i needs to be further explored and clarified. We reviewed the real-world clinical data and the literature on potential mechanisms to gain insight into the pleiotropic effects of emerging antidiabetic agents.

### 3.1 Cardiovascular protection

#### 3.1.1 SGLT2i

##### 3.1.1.1 Clinical trial

SGLT2i have demonstrated superiority over placebo in most cardiovascular outcome trials. In 2019, the DAPA-HF trial, which recruited 4744 patients with HF and reduced ejection fraction, indicated that once-daily dapagliflozin (10 mg) lowered the risk of composite cardiovascular outcomes when compared to placebo (HR, 0.74 [95% CI, 0.65 to 0.85]; *p* < 0.001) (Table 1) (McMurray et al., 2019). Furthermore, in a 2022 trial, dapagliflozin exhibited significant cardioprotective effects (DELIVER) on patients with HF with mild reduced ejection fraction or preserved left ventricular ejection fraction (>40%) (HR, 0.82 [95% CI, 0.73 to 0.92]; *p* < 0.001) (Solomon et al., 2022). These results are consistent with those of the empagliflozin and ertugliflozin outcomes trials in HF with a preserved ejection fraction (EMPEROR-Preserved trial, MK-8835-004 trial) (Cannon et al., 2020; Anker et al., 2021). However, the protective effect of SGLT2i appeared to vary based on gender; a study from Australia indicates that older men with baseline HF benefit more from SGLT2i than women (subdistribution HR, 0.78 [95% CI, 0.66 to 0.93] for men; subdistribution HR, 0.99 [95% CI, 0.77 to 1.28] for women). On the contrary, SGLT2i were observed to improve the outcomes of women with baseline ASCVD (subdistribution HR, 0.98 [95% CI, 0.74 to 0.73] for men; subdistribution HR, 0.36 [95% CI, 0.18 to 0.71] for women) (Sharma et al., 2023). In another study, the effect of canagliflozin on cardiovascular events did not differ by age or sex (HR, 0.71 [95% CI, 0.54 to 0.95] for women; HR, 0.69 [95% CI, 0.56 to 0.84] for men; *p* = 0.8 for interaction) (Yi et al., 2023). The disparate findings of these two reports are intriguing. The absence of beneficial effects of SGLT2i in women with baseline HF may, in part, be attributed to the limited number of women in this specific subgroup. Notably, given the age-related increase in cardiovascular disease (CVD) risk in both genders, particularly among post-menopausal women (Zhao et al., 2018), further investigations focusing on sex differences and involving a substantial number of patients are warranted to ascertain the potential sex-specific benefits of SGLT2i and elucidate the mechanisms involved. In addition, SGLT2i also have a significant advantage in acute HF, suggesting a lower risk of hospitalization (Park et al., 2023). Sotagliflozin has been shown to significantly reduce cardiovascular death-related events in T2DM patients with recent worsening HF (HR, 0.67 [95% CI, 0.52 to 0.85]; *p* < 0.001) (SOLOIST-WHF trial) (Bhatt et al., 2021). Empagliflozin was also associated with a greater reduction in the rate of worsening HF events (HR, 1.36 [95% CI, 1.09 to 1.68]; *p* = 0.0054) (EMPULSE trial) (Voors et al., 2022).

**TABLE 1:** Summary of CV outcome-related trials using SGLT2i, GLP-1RAs, DPP-4i.

Trial	Drug	Study design	Patient characteristics	Treatment dose (median duration)	Primary CV outcome	HR (95%CI), *p*-value
DAPA-HF (McMurray et al., 2019)	Dapagliflozin	randomized, double-blind, placebo-controlled study	Aged>18, NYHA class II, III, or IV symptoms, EF of 40% or less (with or without T2D) (N = 4744)	10 mg/d (18 months)	a composite of worsening HF or death from CV causes	0.74 (0.65–0.85) *p* < 0.001
DELIVER (Solomon et al., 2022)	Dapagliflozin	phase 3, double-blind, randomized, controlled trial	Aged>40, HF and a LVEF of more than 40% (with or without T2D) (N = 6263)	10 mg/d (2.3 years)	worsening HF or CV death	0.82 (0.73–0.92) *p* < 0.001
DECLARE-TIMI 58 (Wiviott et al., 2019)	Dapagliflozin	phase 3, double-blind, randomized, controlled trial	Aged>40, T2D, eGFR ≥60 mL/min also had multiple risk factors for ASCVD or had established ASCVD (N = 17160)	10 mg/d (4.2 years)	MACE (defined as CV death, myocardial infarction, or ischemic stroke). Efficacy outcomes were MACE and a composite of CV death or hospitalization for HF	95%CI < 1.3; *p* < 0.001 for noninferiority, 0.83 (0.73–0.95) *p* = 0.005 for efficacy
EMPEROR-Preserved (Anker et al., 2021)	Empagliflozin	randomized, double-blind, placebo-controlled, event-driven trial	Aged>18, II–IV HF and an EF ≥ 40%, NT-proBNP ≥300 pg/mL (with or without T2D) (N = 5988)	10 mg/d (26.2 months)	a composite of CV death or hospitalization for HF	0.79 (0.69–0.90), *p* < 0.001
MK-8835-004 (Cannon et al., 2020)	Ertugliflozin	double-blind, randomized, placebo-controlled, noninferiority trial	Aged>40, T2D and established ASCVD (N = 8246)	5 or 15 mg/d (3.1 years)	MACE (a composite of death from CV causes, nonfatal myocardial infarction, or nonfatal stroke)	0.97 (0.85–1.11), *p* < 0.001 for noninferiority
SOLOIST-WHF (Bhatt et al., 2021)	Sotagliflozin	phase 3, double-blind, randomized, placebo-controlled trial	aged 18 to 85, T2D and had been hospitalized because of the presence of signs and symptoms of HF and received treatment with intravenous diuretic therapy (N = 1222)	200 mg/d (9.2 months)	the total number of deaths from cardiovascular causes and hospitalizations and urgent visits for HF (first and subsequent events)	0.67 (0.52–0.85), *p* < 0.001
EMPULSE (Voors et al., 2022)	Empagliflozin	randomized, double-blind, placebo-controlled study	with a primary diagnosis of acute denovo or decompensated CHF regardless of LVEF (N = 530)	10 mg/d (3–90 days)	clinical benefit, defined as a hierarchical composite of death from any cause, number of HF events and time to first HF event, or a 5 point or greater difference in change from baseline in the KCCQ-TSS at 90 days	stratified win ratio, 1.36 (1.09–1.68), *p* = 0.0054
ELIXA (Pfeffer et al., 2015)	Lixisenatide	randomized, double-blind, placebo-controlled study	had T2D and had an acute coronary event within 180 days before screening (N = 6068)	10μg–20 μg/d (s.c) (25 months)	death from CV causes, nonfatal myocardial infarction, nonfatal stroke, or hospitalization for unstable angina	1.02 (0.89–1.17), *p* < 0.001 for noninferiority, *p* = 0.81 for surperiority
LEADER (Marso et al., 2016a)	Liraglutide	randomized, double-blind, placebo-controlled study	T2D, aged≥50 (at least one CV condition) or aged≥60 (at least one CV risk factor) (N = 9340)	1.8 mg/d (s.c.) (3.5 years)	the first occurrence of death from CV causes, nonfatal (including silent) myocardial infarction, or nonfatal stroke	0.87 (0.78–0.97), *p* < 0.001 for noninferiority; *p* = 0.01 for superiority
SUSTAIN-6 (Marso et al., 2016b)	Semaglutide	randomized, double-blind, placebo-controlled study	T2D, aged≥50 (established CVD, CHF, or CKD of stage 3 or higher) or aged≥60 (at least one CV risk factor (N = 3297)	0.5/1.0 mg/(s.c.) (2.1 years)	the first occurrence of death from CV causes, nonfatal myocardial infarction (including silent), or nonfatal stroke	0.74 (0.58–0.95), *p* < 0.001 for noninferiority; *p* = 0.02 for superiority
PIONEER6 (Husain et al., 2019)	Semaglutide	randomized, double-blind, placebo-controlled study	T2D, aged≥50 (established CVD, CHF, or CKD of stage 3 or higher) or aged≥60 (at least one CV risk factor) (N = 3183)	14 mg/d (oral) (15.9 months)	the first occurrence of MACE, a composite of death from CV causes, nonfatal myocardial infarction, or nonfatal stroke	0.79 (0.57–1.11), *p* < 0.001 for noninferiority
EXSCEL (Holman et al., 2017)	Exenatide	randomized, double-blind, placebo-controlled study	T2D, had previous CV events (70%), would not have had previous CV events (30%) (N = 14752)	2 mg once weekly (s.c.) (3.2 years)	first occurrence of any composite outcome of death from CV causes, nonfatal myocardial infarction, or nonfatal stroke	0.91 (0.83–1.00), *p* < 0.001 for noninferiority, *p* = 0.06 for superiority
Harmony Outcomes (Hernandez et al., 2018)	Albiglutide	randomized, double-blind, placebo-controlled study	T2D, ≥40, established disease of the coronary, cerebrovascular, or peripheral arterial circulation (N = 9463)	30–50 mg once weekly (s.c.) (1.5 years)	the first occurrence of cardiovascular death, myocardial infarction, or stroke	0·78 (0·68–0·90), *p* < 0·0001 for noninferiority, *p* = 0·0006 for superiority
REWIND (Gerstein et al., 2019)	Dulaglutide	randomized, double-blind, placebo-controlled study	T2D, aged≥50 had to have vascular disease; aged≥55 had to have MI, or lower extremity artery stenosis exceeding 50%, LVH, eGFR <60 mL/min/1.73m^2^, aged≥60 had to have at least two of tobacco use, dyslipidaemia (N = 9901)	1.5 mg once weekly (s.c.) (5.4 years)	the first occurrence of any component of the composite outcome, which comprised non-fatal myocardial infarction, non-fatal stroke, and death from cardiovascular causes or unknown causes	0.88 (0·79 to 0·99), *p* = 0·026
EXAMINE (White et al., 2013)	Alogliptin	randomized, double-blind, placebo-controlled study	T2D and had had an acute coronary syndrome within 15–90 days before randomization (N = 5380)	6.25–25 mg/d (533 days)^a^	a composite of death from CV causes, nonfatal myocardial infarction, or nonfatal stroke	0.96 (≤1.16), *p* < 0.001 for noninferiority *p* = 0.32 for superiority
SAVOR-TIMI53 (Scirica et al., 2013)	Saxagliptin	phase 4, double-blind, randomized, placebo-controlled trial	T2D, ≥55 (men); ≥60 (women)and either a history of established CVD or multiple risk factors for vascular disease (N = 16492)	5 mg/d (2.1 years)	a composite of CV death, nonfatal myocardial infarction, or nonfatal ischemic stroke	1.00 (0.89–1.12), *p* = 0.99 for superiority, *p* < 0.001 for noninferiority
TECOS (Green et al., 2015)	Sitagliptin	randomized, double-blind, placebo-controlled study	T2D with established CVD and were aged≥50 when treated with stable oral anti-hyperglycemic agents or insulin (N = 14671)	50–100 mg/d (3.3 years)^#^	a composite of CV death, nonfatal myocardial infarction, nonfatal stroke, or hospitalization for unstable angina	0.98 (0.88–1.09), *p* < 0.001 for noninferiority, *p* = 0.65 for superiority
CARMELINA (Rosenstock et al., 2019)	Linagliptin	randomized, double-blind, placebo-controlled study	T2D, HbA1c values of 6.5%–10.0% inclusive, and high cardiovascular and renal risk (N = 6979)	5 mg/d (oral) (2.2 years)	the time to first occurrence of CV death, nonfatal myocardial infarction, or nonfatal stroke	1.02 (0.89–1.17), *p* < .001 for noninferiority, *p* = 0.74 for superiority

EF: ejection fraction, LVEF: left ventricular ejection fraction, HbA1c: Hemoglobin A1c, CHF: chronic heart failure, LVH: left ventricular hypertrophy, MI: myocardial ischaemia, KCCQ-TSS: kansas city cardiomyopathy questionnaire total symptom score, ASCVD: atherosclerotic cardiovascular disease, CVD: cardiovascular disease, MACE: major adverse cardiovascular events, HF: heart failure, CV: cardiovascular, NYHA: new york heart association, T2D: Type 2 Diabetes.

^a^ 25 mg/d for GFR≥60 mL/min/1.73 m^2^, 125 mg for GFR, of 30–60 mL/min/1.73 m^2^, 625 mg for GFR ≤30 mL/min/1,73 m^2^; # 50 mg/d for eGFR, was ≥30 and <50 mL/min/1.73 m^2^.

##### 3.1.1.2 Basic research

SGLT2i exhibit superior efficacy in improving cardiovascular diseases, regardless of the presence of diabetes. *In vivo* experiments revealed that dapagliflozin reduced interleukin (IL)-1β expression and can downregulate the activity of [Na^+^] and [Ca^2+^]-related ion channels to alleviate mitochondrial reactive oxygen species, thereby improving angiotensin Ⅱ (Ang Ⅱ)-induced diabetic cardiomyopathy in *db/db* mice (Table 3) (Arow et al., 2020). Consistently, an *in vivo* study focusing on diabetic cardiomyopathy in T1DM rats revealed that dapagliflozin markedly reduced oxidative stress (Rosa et al., 2022). Notably, SGLT2i can restore and maintain sinus rhythm after ablation of atrial fibrillation in T2DM patients (Abu-Qaoud et al., 2023). Interestingly, empagliflozin was found to block the binding of CpG islands in the promoter regions of nuclear factor kappa-B (NF-κB) and superoxide dismutase 2 (SOD2) to ten-eleven translocation (TET2) in cardiomyocytes under high glucose conditions, preventing gene demethylation and alleviating myocardial injury (Scisciola et al., 2023).

**FIGURE 1:**
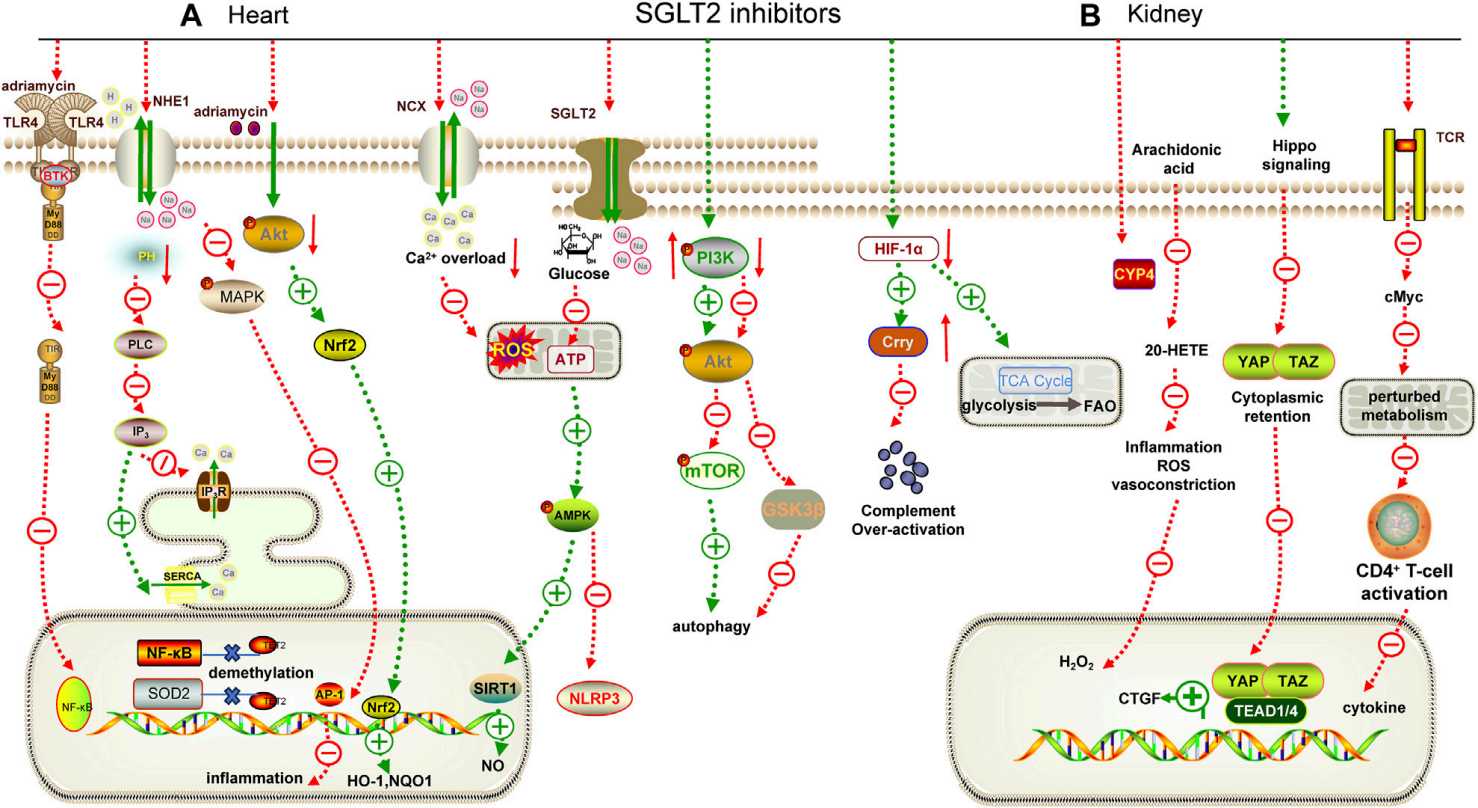
Mechanisms underlying the cardiorenal effects of SGLT2 inhibitors. **(A)** for heart; **(B)** for kidney. AMPK: adenosine monophosphate-activated protein kinase, TLR4: Toll-like receptor 4, MyD88: myeloid differentiation factor 88, NF-κB: nuclear factor-κB, PLC: phospholipase C, IP_3_: inositol 1,4,5-triphosphate, IP_3_R: inositol 1,4,5-triphosphate receptor, SERCA: sarcoplasmic reticulum Ca^2+^-ATPase, MAPK: mitogen-acticated protein kinase, AP-1: activator protein-1, Nrf2: nuclear factor erythroid2-related factor 2, SIRT1: sirtuin 1, PI3K: phosphatidylinositol 3-kinase, mTOR: mammalian target of rapamycin, GSK3β: glycogen synthase kinase 3β, HIF-1α: hypoxia-inducible factor 1-alpha, TCR: T cell receptor, CTGF: connective tissue growth factor, YAP: yes associated protein 1, TAZ: transcriptional coactivator PDZ-binding motif, RTK: receptor tyrosine kinase, NOQ1: NADH dehydrogenase quinone 1, SOD2: superoxide dismutase 2.

In addition to their protective effect against diabetes-induced cardiomyopathy, SGLT2i also have a beneficial effect on other cardiovascular diseases, including ASCVD, which is primarily caused by hypertension-induced inflammation. SGLT2 receptor expression is present in macrophages, which are major players in the inflammatory response. Adenosine 5′-monophosphate-activated protein kinase (AMPK) is a key energy regulator that inhibits the pro-inflammatory effects of macrophages (Packer, 2020a; Jansen et al., 2020). In an atherosclerosis model characterized by a high-fat diet, empagliflozin inhibited NF-κB expression in plaque and reduced the viability of macrophages in *Apoe* ^*−/−*^ mice. Most importantly, it was able to restore p-AMPK expression in macrophages (Fu et al., 2022). Heme oxygenase-1 (HO-1) can protect the cardiovascular system by increasing the bioavailability of NO in endothelial cells. Canagliflozin can increase the expression of HO-1 in endothelial cells and attenuate the adhesion of monocytes to endothelial cells (Peyton et al., 2022). Vascular calcification is a common pathological process in ASCVD. Chen *et al.* demonstrated for the first time that canagliflozin could reduce vascular smooth muscle cells (VSMCs) calcification by down-regulating the expression of NOD-like receptor thermal protein domain associated protein 3 (NLRP3) (Chen et al., 2023). There are many ion channels in cardiomyocytes, such as Na^+^/H^+^ exchanger 1 (NHE1) and Na^+^/Ca^2+^ exchanger (NCX). Pathological conditions that lead to excessive activation or inhibition of ion channels significantly impact the systolic and diastolic movements of the heart. Various hormones (Ang Ⅱ, aldosterone) released during HF or myocardial ischemia (MI) can activate NHE1, inhibit NCX, and lead to intracellular calcium overload, which in turn activates NHE1 and exacerbates calcium overload (Figure 1A) (Kim et al., 2017). Studies have shown that dapagliflozin, empagliflozin, and canagliflozin can inhibit NHE1 to improve endothelial permeability induced by mechanical stretch (Li X. et al., 2021). These studies confirmed that SGLT2i can protect both static and dynamic endothelial cell function (Juni et al., 2021).

Notably, dapagliflozin also inhibits the mitogen-acticated protein kinase/activator protein-1 (MAPK/AP-1) pathway in an NHE1-dependent way to alleviate obesity-induced myocardial inflammation (Lin et al., 2022). Similarly, empagliflozin can improve myocardial injury in obese mice by regulating the AMPK/mammalian target of rapamycin (mTOR) pathway to maintain redox balance (Sun et al., 2020). In addition, empagliflozin also inhibited the overstimulated autophagy in cardiomyocytes by inhibiting the AMPK/glycogen synthase kinase 3β (GSK3β) pathway and NHE1 (Chung et al., 2023; Madonna et al., 2023). These findings highlight the multiple mechanisms by which SGLT2i contribute to the reduction of obesity-related myocardial complications.

SGLT2i also offer promising insights into preventing cardiac toxicity associated with antineoplastic agents. For example, dapagliflozin alleviated adriamycin-induced myocardial injury by inhibiting the phosphoinositide 3-kinase (PI3K)/protein kinase B (PKB)/nuclear factor erythroid 2-related factor 2 (Nrf2) pathway (Hsieh et al., 2022). Empagliflozin could significantly enhance the adriamycin-induced reduction of cardiomyocyte viability and inhibit the expression of NLRP3 and myeloid differentiation factor 88 (MyD88) (Quagliariello et al., 2021). Furthermore, empagliflozin was found to ameliorate sunitinib- and trastuzumab-induced cardiovascular complications by regulating the AMPK/mTOR pathway and ferroptosis (Ren et al., 2021; Min et al., 2023).

In conclusion, the cardiovascular protective effects of SGLT2i in relation to diabetes have been extensively explored. Additionally, SGLT2i have also shown multiple protective mechanisms in animal models of ASCVD, and it has been applied for the treatment of HF (Heidenreich et al., 2022). However, there is still new potential for clinical translation. SGLT2i have demonstrated remarkable advantages in obesity-related, antibiotic-induced, and antineoplastic drug-induced cardiotoxicity (Ren et al., 2021; Min et al., 2023). It is worth noting that although canagliflozin can exhibit anti-inflammatory effects in endothelial cells, recent reports have raised concerns about its specific impact on endothelial cells, which may elevate the risk of amputation (Peyton et al., 2022). Therefore, establishing SGLT2i as routine therapy for diseases beyond diabetes still has a way to go.

#### 3.1.2 GLP-1RAs

##### 3.1.2.1 Clinical trial

Although the previous ELIXA trial did not show superiority of lixisenatide in reducing the rate of major adverse cardiovascular events (MACE) (Table 1) (Pfeffer et al., 2015; Husain et al., 2019), GLP-1RAs has been gradually shown its advantage in improving cardiovascular outcomes in recent trials. Cardiovascular mortality among patients with T2DM and high cardiovascular risk was found to be lower with liraglutide than with placebo (HR, 0.87 [95% CI, 0.78 to 0.97]; *p* = 0.01) (LEADER) (Marso et al., 2016a). The SUSTAIN-6 trial, which involved 3,297 patients with T2DM and high cardiovascular risk, found that twice-weekly semaglutide significantly reduced the incidence of MACE (HR, 0.74 [95% CI, 0.58 to 0.95]; *p* = 0.02) (Marso et al., 2016b). In the Harmony Outcomes trial, the rate of MACE in T2DM patients with the addition of albiglutide (30–50 mg once-weekly) was lower than the placebo group (HR, 0.78 [95% CI, 0.68 to 0.90]; *p* = 0.0006) (Hernandez et al., 2018). Lastly, the REWIND trial revealed a reduction in MACE with once-weekly dulaglutide in T2DM patients (HR, 0.88 [95% CI, 0.79 to 0.99]; *p* = 0.026) (Gerstein et al., 2019).

##### 3.1.2.2 Basic research

**FIGURE 2:**
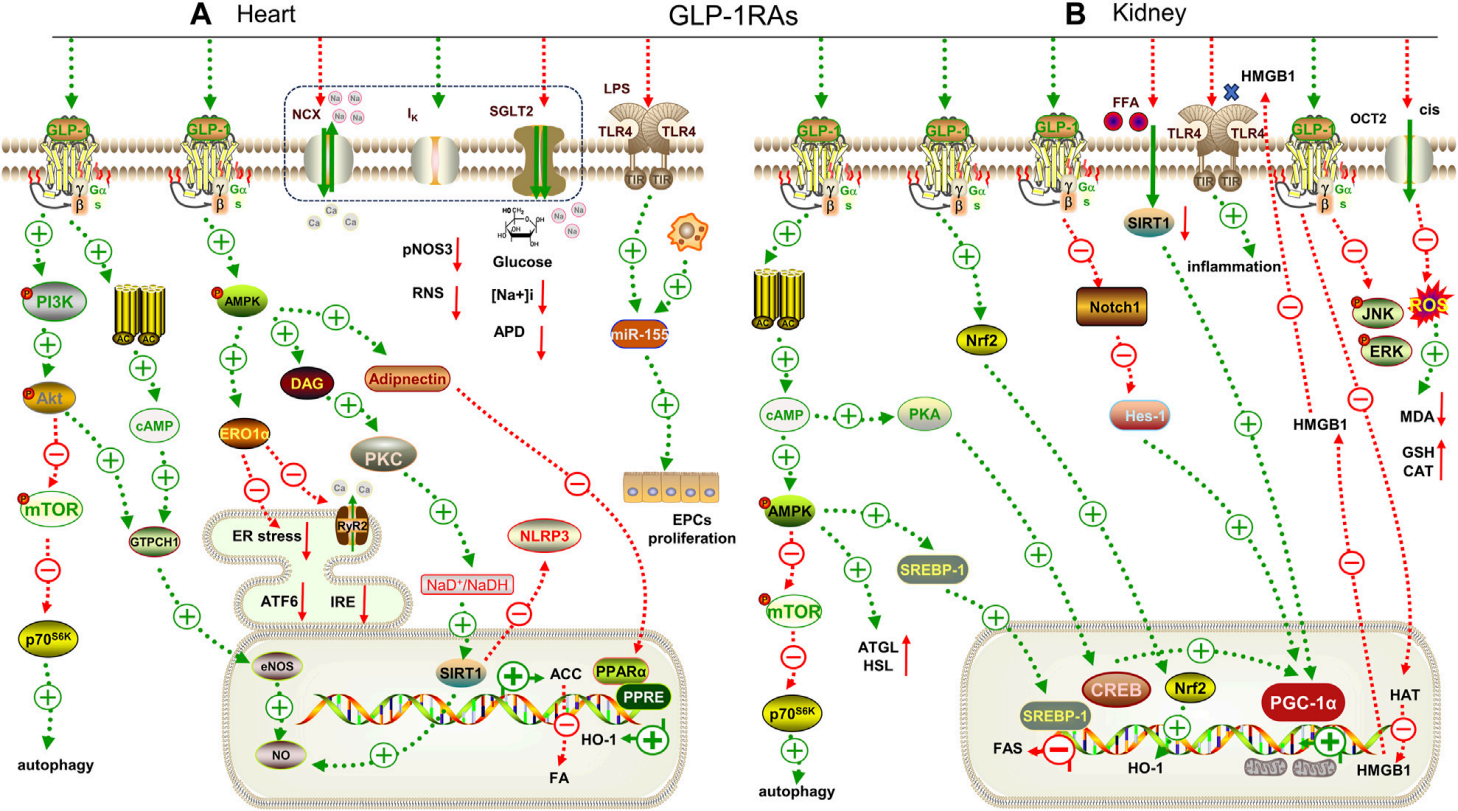
Mechanisms underlying the cardiorenal effects of GLP-1RAs. **(A)** for heart; **(B)** for kidney. GTPCH1: GTPcyclohydrolase Ⅰ, eNOS: endothelial nitric oxide synthase, ERO1α: endoplasmic oxidoreductin 1 like protein, ATF6: activating transcription factor 6, IRE: inositol-requiring enzyme, RyR2: ryanodine receptor 2, DAG: diacylglycerol, PPARα: peroxisome proliferator-activated receptor α, ATGL: adipose triglyceride lipase, HSL: hormone-sensitive lipase, SREBP-1: sterol regulatory element binding protein-1, FAS: fatty acid synthase, CREB: cAMP-response element binding protein, PGC-1α: peroxisome proliferator-activated receptor gamma coactivator-1alpha, Notch1: Notch homolog 1, Hes-1: hairy and enhancer of split-1, MDA: malondialdehyde. GSH: glutathione. pNOS3: phospho-endothelial nitric oxide, RNS: nitrogen species, APD: action potential duration, HMGB1: high mobility group protein 1, CAT: catalase, HAT: histone acrtyltransferases, LPS: lipopolysaccharide, ACC: acetyl CoA carboxylase.

Extensive basic studies overwhelmingly support the observed beneficial effects of GLP-1RAs in clinical trials. Diabetes is often accompanied by lipid metabolism disorders, causing mitochondrial dysfunction. Studies have shown that liraglutide can inhibit the diacylglycerol/protein kinase C (DAG/PKC) pathway by activating AMPK, and upregulate Sirtuin 1 (SIRT1) to inactivate acetyl-CoA carboxylase phosphorylation, thereby reducing lipid-overloaded cardiomyocyte injury in streptozotocin-induced diabetic rats (Figure 2A) (Table 3)(Inoue et al., 2015). Similarly, liraglutide also increased adiponectin secretion and restored peroxisome proliferator-activated receptor gamma coactivator-1α (PGC-1α) expression by upregulating AMPK, which ameliorated IL-1β-induced mitochondrial damage in HL-1 cells (Zhang et al., 2020). Dulaglutide reduced the expression of NLRP3, IL-1β, and endoplasmic reticulum stress-related proteins induced by high glucose in human umbilical vein endothelial cells (HUVECs) via upregulating SIRT1 (Luo et al., 2019). Moreover, liraglutide enhanced the angiogenic potential of CD34 hematopoietic stem cells under high glucose conditions by activating the protective PI3K/PKB pathway and stimulating mitochondrial respiration (Sforza et al., 2022). Exenatide protects against high glucose-induced myocardial injury by inhibiting the NF-κB pathway and reducing the expression of tumor necrosis factor α (TNF-α) and monocyte chemotactic protein-1 (MCP-1) (Fu et al., 2020).

GLP-1RAs exhibit similar potential to SGLT2i in improving ASCVD. Liraglutide has been shown to attenuate plaque formation in *Apoe* ^*−/−*^ mice by inducing cell cycle arrest in VSMCs in an AMPK-dependent or AMPK-independent manner (Jojima et al., 2017; Koshibu et al., 2019). Moreover, in the same mouse model, liraglutide was able to induce plaque regression by modulating bone marrow-derived macrophages to convert to anti-inflammatory phenotypes in the established plaque (Bruen et al., 2019).

GLP-1RAs have also demonstrated their potential in improving various other types of heart disease, including obesity-related, senile, and inflammatory heart disease, as well as improving the function of donor hearts after isolation. For example, liraglutide alleviates vascular inflammation in obesity by upregulating pAMPK expression and promoting Nrf2 nuclear translocation (Liu et al., 2023). It has also been shown to restore autophagy by inhibiting the mTOR/phosphoprotein 70 ribosomal protein S6 kinase (p70S6K) pathway caused by abdominal aortic coarctation (Zheng et al., 2020), regulate iNCX, delayed after potassium channel (I_k_) and ryanodine receptor 2 (RyR2) channels in the myocardium, and restore mitochondrial membrane depolarization to protect the aged heart (Durak and Turan, 2023). Recent studies have also shown that acute administration of exenatide can increase NO to maintain good diastolic function after reperfusion in isolated hearts (Kadowaki et al., 2023). Furthermore, semaglutide can reduce lipopolysaccharides (LPS)-induced miR-155 secretion from macrophage exosomes to protect endothelial progenitor cell function (Pan et al., 2023). These findings highlight the multifaceted beneficial effects of GLP-1RAs on the cardiovascular system.

Currently, the superiority of GLP-1RAs in cardiovascular protection is primarily focused on diabetes-related conditions, as demonstrated above. However, recent findings have revealed that apart from their hypoglycemic effects, GLP-1RAs also have therapeutic efficacy in weight loss (Campbell et al., 2023). Basic research show their potential anti-inflammatory effects in obesity-related heart disease and advantages in ASCVD and aged animal models (Withaar et al., 2023). Despite the lack of clinical evidence, these findings suggest that GLP-1RAs may be used as a second or third-line therapy for non-diabetes-related cardiovascular diseases in the future.

#### 3.1.3 DPP-4i

##### 3.1.3.1 Clinical trial

Most of the cardiovascular outcome trials of DPP-4i have demonstrated cardiovascular safety rather than superiority. The EXAMINE trial, which included 5380 T2DM patients with a recent acute coronary event, showed that the rate of MACE with the addition of alogliptin was not superior to that of placebo (HR, 0.96 [upper boundary of the one-sided repeated CI, 1.16]; *p* < 0.001 for non-inferiority) (Table 1) (White et al., 2013). In the SAVOR-TIMI 53 trial, saxagliptin did not increase the rate of MACE in the elderly and very elderly patients (HR, 1.00 [95% CI, 0.89 to 1.12]; *p* = 0.99), but saxagliptin was surprisingly associated with an increased risk for HF hospitalization, thus its use requires detailed evaluation (HR, 1.27 [95% CI, 1.07 to 1.51]; *p* = 0.007) (Scirica et al., 2013; Leiter et al., 2015). The TECOS trial showed that sitagliptin did not increase the rate of MACE and hospitalizations for HF in patients with T2DM, even in high-risk patients (HR, 0.98 [95% CI, 0.88 to 1.09]; *p* = 0.65) (Green et al., 2015; McGuire et al., 2016). Similarly, sitagliptin was not superior to placebo in terms of efficacy in T2DM patients with ASCVD (Nauck et al., 2019). The CARMELINA trial indicated that linagliptin added to usual treatment resulted in a non-inferior risk of MACE in T2DM patients with high cardiovascular risk (HR, 1.02 [95% CI, 0.89 to 1.17]; *p* = 0.74) (Rosenstock et al., 2019). However, a trial from Thailand showed that linagliptin was superior in reducing 10-year cardiovascular risk score in patients with a baseline risk greater than 20%, with enhanced outcomes in older patients (Poonchuay et al., 2022).

##### 3.1.3.2 Basic research

**FIGURE 3:**
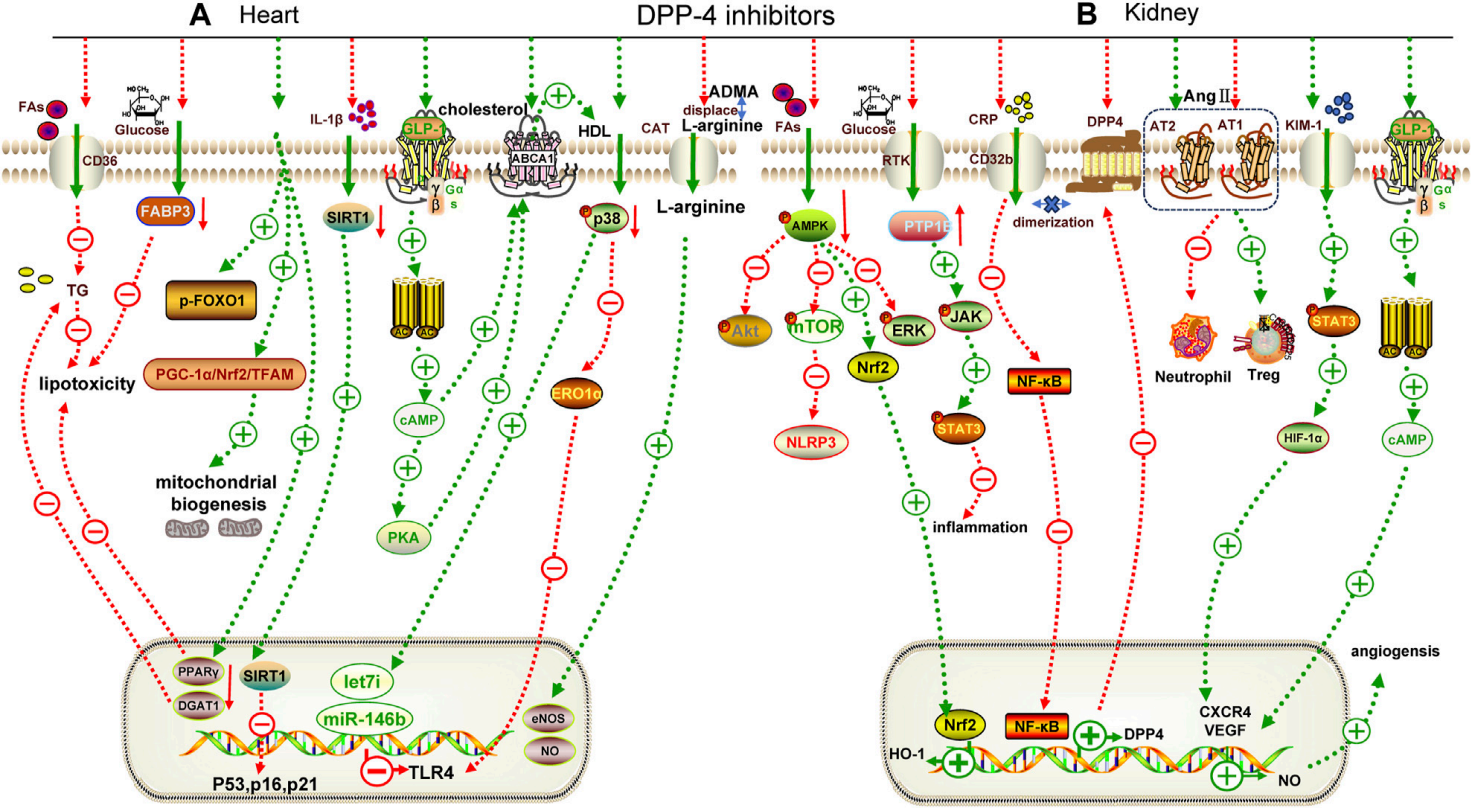
Mechanisms underlying the cardiorenal effects of DPP-4 inhibitors. **(A)** for heart; **(B)** for kidney. TG: triglyceride, FABP3: fatty acid binding protein 3, p-FOXO1: phospho-Forkhead box protein O1, ABCA1: adenosine triphosphate transporter A1, PTP1B: protein tyrosine phosphatase, CRP: C-reactive protein, KIM-1: kidney injury molecule 1, CXCR4: chemokine receptor 4, AT1: angiotensin receptor 1, AT2: angiotensin receptor 2, ERK: extracellular regulated protein kinase, DGAT1: diacylgycerol acyltransferase 1, CAT: cationic amino acid transporters, HDL: high density lipoprotein, TFAM: mitochondrial transcription factor A, eNOS: endothelial nitric oxide synthase, DGAT1: diacylglycerol o-acyltransferase 1, PPARγ: peroxisome proliferator activated receptor γ, JAK: janus tyrosine kinase, STAT3: signal transducer and activator of transcription 3.

While clinical trials have not demonstrated significant advantages of DPP-4i, basic research has revealed their cardioprotective effect. The normal diastolic and systolic functions of the heart depend on the energy provided by a large number of mitochondria and fatty acid oxidation in cardiomyocytes. Evogliptin (an oral hypoglycemic drug approved for the treatment of T2DM in South Korea in 2015) can restore the expression of the mitochondrial-synthesis-related pathway, PGC-1α/Nrf2/mitochondrial transcription factor A (TFAM), to promote normal mitochondrial synthesis (Gureev et al., 2019) and inhibit the expression of lipid transmembrane transporters (fatty acid binding protein 3, FABP3) (Zhang et al., 2015) and synthetic proteins (Forkhead box protein O1, FOXO1; peroxisome proliferator activated receptor γ, PPARγ; diacylglycerol o-acyltransferase 1, DGAT1) (Kyriazis et al., 2021) to block the over-activated lipid pathway in *db/db* mice (Figure 3A) (Pham et al., 2023). Further studies have shown that sitagliptin combined with insulin can improve diabetic cardiomyopathy by reducing the expression of inflammatory factors to a greater extent (Table 3) (Wadie et al., 2022).

In addition, DPP-4i have shown significant advantages in non-diabetic cardiovascular diseases, particularly ASCVD. Trelagliptin inhibits IL-1β-induced MCP-1 expression in human aortic endothelial cells by inhibiting the NF-κB pathway, preventing monocyte infiltration during atherosclerosis (Meng et al., 2020). Alogliptin has been shown to reduce IL-1β-induced inflammatory cytokine expression in VSMCs by restoring SIRT1 expression and downregulating senescence-related markers (p16, p21 and p53) to prevent premature smooth muscle cell senescence and enhance plaque progression (Zhao et al., 2021). Reverse cholesterol transport is an important mechanism for improving ASCVD, which is mainly mediated by high-density lipoprotein (HDL)-associated cyclic adenosine monophosphate (cAMP) and can activate adenosine triphosphate transporter A1 (ABCA1) to promote HDL formation. Sitagliptin increases intracellular cAMP levels by indirectly activating GLP-1R and up-regulating ABCA1 expression, thereby promoting reverse cholesterol transport in macrophages and reducing foam cell generation (Komatsu et al., 2023). In an MI model of *db/db* mice, linagliptin upregulated the expression of microRNAs (miR-146b and Let-7i) in cardiomyocytes by reducing p38 phosphorylation, thereby inhibiting toll-like receptor 4 (TLR4) upregulation (Birnbaum et al., 2019).

DPP-4i also offer benefits in improving metabolic syndrome. Plasma asymmetrical dimethylarginine (ADMA) is increased in fructose-induced metabolic syndrome, which can inhibit NO by replacing L-arginine, the substrate of NO synthase, and aggravate endothelial dysfunction. Sitagliptin reduces endothelial dysfunction by increasing the activity of dimethylarginine dimethylaminohydrolase 1 (DDAH1) (an enzyme that degrades ADMA) in the kidney to degrade ADMA, thereby increasing plasma NO levels (Wójcicka et al., 2023).

Although published clinical trials have not demonstrated the superior cardiovascular benefits of DPP-4i in diabetic population, extensive basic researches have highlighted its protective effects on the cardiovascular system in animal models of metabolic syndrome, ASCVD, and MI. These findings emphasize the necessity for further exploration into the potential clinical application of DPP-4i.

### 3.2 Renal protection

#### 3.2.1 SGLT2i

##### 3.2.1.1 Clinical trial

SGLT2i have demonstrated significant advantages in kidney- and cardiovascular system-related clinical trials. Dapagliflozin has been shown to reduce HF hospitalization rates in T2DM patients (DECLARE-TIMI 58) (HR, 0.83 [95% CI, 0.73 to 0.95]; *p* = 0.005) (Wiviott et al., 2019). In the DAPA-CKD trial, dapagliflozin exhibited superior efficacy in mitigating sustained eGFR decline of at least 50% in both diabetic and non-diabetic patients with CKD (Table 2) (HR, 0.61 [95% CI, 0.51 to 0.72]; *p* < 0.001) (Heerspink et al., 2020). The analysis of the DAPA-CKD trial also demonstrated the superior benefits of dapagliflozin in reducing albuminuria and improving eGFR in T2DM patients (Heerspink et al., 2021; Jongs et al., 2021). In addition, the analysis of the DELIVER trial (Solomon et al., 2022) also showed that dapagliflozin significantly slowed the decline in eGFR from the baseline (difference in eGFR decline from baseline was 0.5, [95% CI, 0.1–0.9 mL/min/1.73 m^2^ per year]; *p* = 0.01) (Mc Causland et al., 2023). However, the failure of dapagliflozin to improve GFR in the DIAMOND trial, which focused on patients with non-T2DM CKD (difference in mean proteinuria change from baseline was 0.9%, [95% CI, −16.6 to 22.1]; *p* = 0.93), suggests that the specific effects of dapagliflozin on GFR need to be further investigated (Cherney et al., 2020). In the CREDENCE trial, canagliflozin significantly reduced the rates of end-stage renal disease (ESRD) and doubled serum creatinine (HR, 0.70 [95% CI, 0.59 to 0.82]; *p* = 0.00001) (Perkovic et al., 2019). Lastly, empagliflozin (10 mg/day) was significantly superior to placebo in slowing kidney disease in the EMPA-KIDNEY trial (HR, 0.72 [95% CI, 0.6 to 0.82]; *p* < 0.001) (Herrington et al., 2023).

**TABLE 2:** Summary of renal outcome-related trials using SGLT2i, GLP-1RAs, DPP-4i.

Trial	Drug	Study design	Patient characteristics	Treatment dose (median duration)	Primary renal outcome	HR (95%CI), *p*-value
DAPA-CKD (Heerspink et al., 2020)	Dapagliflozin	randomized, double-blind, placebo-controlled study	with or without T2D who had an eGFR of 25–75 mL/min/1.73m^2^ and a UACR of 200–5000(N = 4744)	10 mg once daily (2.4 years)	The first occurrence of any of the following: a decline of at least 50% in the eGFR (confirmed by a second Scr measurement after ≥28 days), the onset of ESKD (defined as maintenance dialysis for ≥28 days, kidney transplantation, or an eGFR of <15 mL/min/1.73 m^2^ confirmed by a second measurement after ≥28 days), or death from renal or cardiovascular causes	0.61 (0.51–0.72), *p* < 0.001
DIAMOND (Cherney et al., 2020)	Dapagliflozin	randomized, double-blind, placebo-controlled study	aged 18–75 years, with CKD, without T2D, with a 24 h urinary protein excretion >500–3500 mg, eGFR≥25 mL/min/1.73m^2^, and who were on stable RAS blockade (N = 58)	10 mg/d (treat 6 weeks with 6-week washout in between)	The percentage change from baseline in 24 h proteinuria during dapagliflozin treatment relative to placebo	dapagliflozin *versus* placebo was −6·6 mL/min/1·73 m^2^ (−9·0 to −4·2; *p* < 0·0001)
CREDENCE (Perkovic et al., 2019)	Canagliflozin	randomized, double-blind, placebo-controlled study	aged ≥30, had T2DM, also required to have CKD (defined as an eGFR of 30 to <90 mL/min/1.73m^2^), UACR>300 to 5000(N = 4401)	100 mg once daily (2.62 years)	A composite of ESKD, doubling of the Scr level from baseline (average of randomization and pre-randomization value) sustained for at least 30 days according to central laboratory assessment, or death from renal or cardiovascular disease	0.70 (0.59–0.82), *p* = 0.00001
EMPA-KIDNEY (Herrington et al., 2023)	Empagliflozin	randomized, double-blind, placebo-controlled study	with or without T2DM, eGFR of at least 20 but less than 45 mL/min/1.73m^2^, regardless of the level of albuminuria, or with an eGFR of at least 45 but less than 90 mL/min/1.73m^2^ with UACR of at least 200 at the screening visit (N = 6609)	10 mg once daily (2 years)	The first occurrence of ESRD or death from cardiovascular causes; the initiation of maintenance dialysis or receipt of a kidney transplant, a sustained decrease in the eGFR to less than 10 mL/min/1.73m^2^, a sustained decrease from baseline in the eGFR of at least 40%, or death from renal causes	0.72 (0.6–0.82), *p* < 0.001
LEADER (Mann et al., 2017) prespecified secondary analysis	Liraglutide	randomized, double-blind, placebo-controlled study	aged ≥50, T2D with at least one cardiovascular coexisting condition or an age of 60 years or more with at least one cardiovascular risk factor (N = 9340)	1.8 mg once daily (s.c.) (3.5 years)	The composite renal outcome consisted of new-onset persistent macroalbuminuria, persistent doubling of the serum creatinine level and an eGFR of 45 or less mL/minute/1.73m^2^, the need for continuous RRT with no reversible cause of the renal disease, or death from renal disease	0.78 (0.67–0.92), *p* = 0.003
AMPLITUDE-O (Gerstein et al., 2021)	Efpeglenatide	randomized, double-blind, placebo-controlled study	aged ≥18, T2D, had a history of cardiovascular disease or ≥50 (men); ≥55 (women) and had kidney disease defined as an eGFR of 25.0–59.9 mL/min/1.73m^2^, and at least one additional cardiovascular risk factor (N = 4076)	2 mg/week for 4 weeks, then 4 mg/week for 4 weeks, and then 6 mg/week until the end (1.81 years)	A composite renal outcome incident macroalbuminuria defined as a UACR>300, plus an increase in the UACR of ≥30% from baseline, a sustained decrease in the eGFR of ≥40% for ≥30 days, renal-replacement therapy for ≥90 days, or a sustained eGFR of <15 mL/min/1.73m^2^ for ≥30 days	0.68 (0.57–0.79), *p* < 0.001
FLOW (Rossing et al., 2023)	Semaglutide	phase 3b, randomized, double-blind, placebo-controlled study	aged ≥18 years or ≥20 years in Japan with pre-existing CKD with high albuminuria, low eGFR, T2D, HbA1c ≤ 10% (<86 mmol/mol) and on stable treatment with the maximum labelled or tolerated dose of a RAAS blocking agent (N = 3534)	0.25 mg/week for 4 weeks, then 0.5 mg/week for 4 weeks, and then 1.0 mg/week until the end (s.c.)	Ongoing	Ongoing
GUARD (Yoon et al., 2017)	Gemigliptin	randomized, double-blind, placebo-controlled study	aged 19–75 years, diagnosed with T2D, and confirmed to have moderate (eGFR: 30–59 mL/min/1.73m^2^) to severe (eGFR: 15–29 mL/min/1.73m^2^) (N = 132)	50 mg daily (12 weeks)	Changes in eGFR, UACR at Week 12	gemigliptin group, the mean decrease in UACR was significant, MA (−41.9 mg/g creatinine, *p* = 0.03) and macroalbuminuria (−528.9 mg/g creatinine, *p* < 0.001)
GUARD-extension (Han et al., 2018)	Gemigliptin	randomized, double-blind, placebo-controlled study	Patients who had completed the 12-week study and consented to participate in the extended study were enrolled. (N = 102)	50 mg of gemigliptin daily; 5 mg of linagliptin daily	Changes in eGFR, UACR at Week 52	eGFR decreased by 3.86 mL/min/1.73m^2^ in the gemigliptin group and 1.85 mL/min/1.73m^2^ in the placebo/linagliptin group. The UACR did not change significantly in either group between baseline and week 52

UACR: urine albumin creatine ratio, CKD: chronic kidney disease, eGFR: estimated Glomerular Filtration Rate, ESKD: End-Stage Kidney Disease, T2D: Type 2 Diabetes, MA: microalbuminuria, RAS: renin angiotensin system, SCr: Serum Creatine rate, RRT: renal replacement therapy.

##### 3.2.1.2 Basic research

Promising results have been obtained from basic research on the role of SGLT2i in alleviating kidney injury. High glucose stimulation induces heightened energy consumption in HK-2 cells, leading to a decrease in the intracellular adenosine-diphosphate/adenosine-triphosphate (ADP/ATP) ratio. This disruption affects the AMPK/mTOR pathway, resulting in reduced autophagy and inhibited energy production (Figure 1B) (Xiao et al., 2011; Packer, 2020b). Dapagliflozin may reverse this process (Xu et al., 2021). Cytochrome P4 (CYP4) is highly expressed in diabetic kidney and can metabolize arachidonic acid into 20-hydroxy-eicosapentaenoic acid (20-HETE), which promotes the formation of superoxide. Dapagliflozin can reduce the inflammation of DKD by targeting the CYP4/20-HETE pathway (Dia et al., 2023). In addition, dapagliflozin could appropriately restore fatty acid metabolism to improve the activation of hypoxia-inducible factor-1α (HIF-1α) and metabolite accumulation caused by mitochondrial tricarboxylic acid (TCA) cycle over-activation under DKD, suggesting that SGLT2i could prevent tubular cell metabolic shift and associate with inflammation (Ke et al., 2022). The Hippo-yes associated protein 1/transcriptional coactivator (YAP/TAZ) pathway plays an important role in fibrosis. Dapagliflozin can inhibit the nuclear translocation of YAP/TAZ, thereby reducing the transcription of its downstream pro-fibrotic target genes connective tissue growth factor (CTGF) to improve DKD fibrosis (Table 3) (Feng et al., 2023). Empagliflozin can also delay DKD fibrosis by preventing reprogramming of serine-threonine metabolism (Lu et al., 2022).

**TABLE 3:** Summary of *in vitro* and *in vivo* models using SGLT2i, GLP-1RAs, and DPP-4i.

Drug	Animal treatment	Mice type	Cell type	Mechanism	References
dapagliflozin	Ang Ⅱ (cardiomyopathy)	db/db mice	primary SD rat ventricular myocytes	anti-oxidative stress	Arow et al. (2020)
dapagliflozin	STZ (T1D cardiomyopathy)	Wistar rats	-	anti-oxidative stress	Rosa et al. (2022)
empagliflozin	HFD (atherosclerosis)	Apoe^−/−^ mice	RAW264.7 cell	anti-inflammatory	Fu et al. (2022)
dapagliflozin	HFD (obesity-related cardiac dysfunction)	C57 mice	rat cardiomyocyte H9c2 cells	anti-inflammatory	Lin et al. (2022)
empagliflozin	Isoproterenol (HF)	Wistar rats	human atrial fibroblasts	anti-fibrosis	Chung et al. (2023)
dapagliflozin	DOX (cardiomyopathy)	SD rat	rat cardiomyocyte H9c2 cells	anti-oxidative stress/inflammatory/fibrosis	Hsieh et al. (2022)
empagliflozin	sunitinib (cardiomyopathy)	C57 mice	rat cardiomyocyte H9c2 cells	inhibition of autophagy	Ren et al. (2021)
empagliflozin	trastuzumab (cardiomyopathy)	C57 mice	primary C57 mice myocytes	ferroptosis	Min et al. (2023)
dapagliflozin	STZ + HFD (DKD)	SD rat	HK-2 cell	anti-fibrosis	Feng et al. (2023)
empagliflozin	LPS (acute septic renal injury)	C57 mice	-	anti-inflammatory	(Maayah et al., 2021)
empagliflozin	lupus-prone mice (lupus nephrities)	MRL/lpr mice	podocyte	anti-inflammatory	Zhao et al. (2023)
liraglutide	STZ (T1D cardiomyopathy)	Wistar rats	-	anti-oxidative stress	Inoue et al. (2015)
liraglutide	-	-	hematopoietic stem progenitor cells (HSPCs)	enhanced angiogenic potential	Sforza et al. (2022)
liraglutide	STZ (atherosclerosis)	Apoe^−/−^ mice	human umbilical vein endothelial cells (HUVECs)	anti-inflammatory	Koshibu et al. (2019)
liraglutide	AAC (myocardial fibrosis)	SD rat	-	anti-fibrosis	Zheng et al. (2020)
semaglutide	-	-	endothelial progenitor cells (EPCs)/RAW264.7 cell	anti-inflammatory	Pan et al. (2023)
liraglutide	HFD (DKD)	zucker diabetic fatty rats	Hkc8/HEK293 cells	anti-oxidative stress	Yang et al. (2020)
liraglutide	HSD (DKD)	zucker fatty rats	-	anti-inflammatory	Sukumaran et al. (2019)
exenatide	HFD (obesity-related kidney dysfunction)	C57 mice	HK-2 cell	anti-oxidative stress/apoptosis	Wang et al. (2021)
liraglutide	IRI-AKI	C57 mice	HK-2 cell	anti-inflammatory	Li et al. (2021a)
liraglutide	GM-AKI	SD rat	-	anti-oxidative stress/apoptosis/inflammatory	Elkhoely (2023)
liraglutide	Cis-AKI	SD rat	-	anti-oxidative stress/apoptosis/inflammatory	Sharaf et al. (2023)
sitagliptin	STZ (T1D cardiomyopathy)	SD rat	-	anti-inflammatory	Wadie et al. (2022)
trelagliptin	IL-1β (atherosclerosis)	-	human aortic endothelial cells (HAECs)	anti-inflammatory	Meng et al. (2020)
linagliptin	IRI-MI	db/db mice	primary human cardiofbroblasts (HCF)/cardiomyocytes (HCM)	anti-inflammatory	Birnbaum et al. (2019)
sitagliptin	STZ (DKD)	Wistar rats	-	anti-inflammatory	Al-Qabbaa et al. (2023)
linagliptin	STZ (DKD)	CD-1 mice	human dermal microvascular endothelial cells (HMVECs)	anti-fibrosis	Kanasaki et al. (2014)
saxagliptin	Ang Ⅱ (hypertensive nephropathy)	C57 mice	T35OK-ANG II type 1 A receptor (AT_1A_R) (OK) cells (opossum-derived proximal tubule cells)	anti-inflammatory	Nistala et al. (2021)
saxagliptin	GM-AKI	SD rat	-	anti-oxidative stress/apoptosis/inflammatory	Mayer et al. (2021)

HFD: high-fat diet, HF:heart failure, T1D: type 1 diabete, DOX: doxorubicin, STZ: streptozotocin, LPS: lipopolysaccharide, AAC: abdominal aortic constriction. HSD: high-salt diet, IRI: ischemia-reperfusion injury, GM: gentamicin, Cis: cisplatin, AKI: acute kidney injury, MI: myocardial infarction, DKD: diabetic kidney disease.

In recent years, the role of SGLT2i in mediating immune response has attracted great attention. Canagliflozin has been shown to inhibit CD4^+^T cell activation and reduce cancer myelocytomatosis oncogene (cMyc) to prevent metabolic reprogramming and immune inflammation (Jenkins et al., 2023). Consistently, a study by Zhao et al. revealed that empagliflozin can inhibit the over-activated SGLT2 in lupus kidney glomeruli, prevent the activation of mechanistic target of rapamycin complex 1 (mTORC1), and delay glomerular injury in lupus kidney (Zhao et al., 2023). Also, the expression of complement receptor type 1-related protein y (Crry), a key complement regulator, was upregulated by dapagliflozin, inhibiting HIF-1α accumulation under high glucose to alleviate immune inflammatory injury in *db/db* mice (Chang et al., 2021).

SGLT2i have a significant protective effect on DKD. Immune-related nephropathy is identified as a major contributor to CKD, and several basic studies have confirmed the positive role of SGLT2i in regulating the immune system. As a result, Säemann et al. advocate for the inclusion of patients with autoimmune diseases in large-scale renal outcome trials (Säemann and Kronbichler, 2022). Currently, relevant clinical trials have confirmed the acceptable safety profile of SGLT2i in the treatment of lupus nephritis, but further evaluation is needed to assess its efficacy (Wang et al., 2022).

#### 3.2.2 GLP-1RAs

##### 3.2.2.1 Clinical trial

Most clinical trials of GLP-1RAs have been *post hoc* and prespecified analyses, highlighting their role in reducing eGFR and urine albumin creatine ratio (UACR). A prespecified analysis of renal outcomes in the LEADER trial showed a significant improvement in macroalbuminuria with liraglutide (HR, 0.78 [95% CI, 0.67 to 0.92]; *p* = 0.003) (Table 2) (Mann et al., 2017). In a *post hoc* analysis of the (SUSTAIN1-7) trial, semaglutide has a significant effect on reducing UACR but decreasing eGFR only in an early stage in T2DM patients with established CKD (Mann et al., 2020). Similarly, in the pooled analysis of SUSTAIN 6 and LEADER, both liraglutide and semaglutide reduced albuminuria by 24% over 2 years (95% CI, 20%–27%; *p* < 0.001), with a greater delay in the continuous decline of eGFR at an eGFR of 30–60 mL/min/1.73 m^2^ (Shaman et al., 2022). In addition, a pooled analysis of the SUSTAIN 6 and PIONEER 6 trials showed that although the improvement in eGFR slope was not significant in subgroups, semaglutide still reduced the eGFR slope in an overall population analysis (Tuttle et al., 2023). Of note, in a *post hoc* analysis of the STEP1-3 trial in obese patients, a higher dose (2.4 mg) of once-weekly semaglutide reduced UACR by 20.6%, while there was no difference between semaglutide and placebo in the eGFR slope at week 68 (Heerspink et al., 2023). A direct, specific trial is underway to assess whether semaglutide can delay DKD (FLOW) in older patients who have had T2DM for nearly two decades, which will provide novel insights into the long-term renal effects of GLP-1RAs (Rossing et al., 2023).

##### 3.2.2.2 Basic research

Similar to SGLT2i, GLP-1RAs have shown hopeful results in delaying the progression of kidney disease, regardless of diabetes status. Nrf2 expression was significantly upregulated by liraglutide, activating the AMPK/mTOR pathway and thereby alleviating DKD (Figure 2B) (Table 3) (Yang et al., 2020). Further, Nrf2 can regulate the disorder of lipid metabolism through the AMPK pathway to reduce ectopic lipid deposition in renal tubules in DKD (Su et al., 2020). Notably, co-administration of exenatide and adipose-derived mesenchymal stem cells (ADMSCs) significantly improved the renal function of DKD (Habib et al., 2021).

Additionally, GLP-1RAs may have therapeutic promise in renal injury caused by hypertension, obesity or ischemia reperfusion. Studies have shown that liraglutide can reduce blood pressure by increasing the expression of endothelial nitric oxide synthase (eNOS) and vascular endothelial growth factor (VEGF), thereby improving the vasoconstriction of intrarenal arterioles. It can also reduce the infiltration of macrophages into renal vascular endothelial cells and alleviate renal vascular inflammation in obese rats induced by a high-salt diet (Sukumaran et al., 2019). Exenatide can stabilize mitochondrial membrane potential and reduce palmitate-induced reactive oxygen species production in HK-2 cells through the upregulation of SIRT1 (Wang et al., 2021). High mobility group box 1 protein (HMGB1) is a damage-associated molecular pattern, which is released from the nucleus to the cytoplasm during renal ischemia and then binds to its receptors, such as TLR-4, to promote the inflammatory cascade. Liraglutide can downregulate the expression of HMGB1 receptors and prevent acetylation of HMGB1 by increasing histone acrtyltransferases (HAT) activity, thereby reducing neutrophil infiltration and delaying renal ischemia-reperfusion injury *in vivo* and *in vitro* (Li Y. et al., 2021).

GLP-1RAs have also been shown to have a positive role in reducing the nephrotoxic effects of antibiotics and antitumor drugs. Liraglutide can mediate mitochondrial biogenesis by regulating the protein kinase A/cyclic-AMP response binding protein (PKA/CREB) and notch homolog 1/hairy and enhancer of split-1 (Notch/Hes-1) pathways and up-regulating the expression of PGC-1α to activate Nrf2, thereby improving the nephrotoxicity induced by glucocorticoids (Elkhoely, 2023). Cisplatin, a common and effective chemotherapeutic agent, often causes irreversible acute kidney injury (AKI). Organic cations transporter 2 (OCT2) is located on the basement membrane of renal tubules and is responsible for the absorption of cisplatin. The MAPK pathway plays a pivotal role in cisplatin-induced AKI. Liraglutide can reduce renal injury by inhibiting the expression of OCT2 and c-Jun N-terminal kinase/extracellular regulated protein kinase (JNK/ERK), thereby restoring the oxidative/antioxidant balance (Sharaf et al., 2023). Additionally, liraglutide also inhibited the release of HMGB1 to reduce cisplatin-induced apoptosis in HK-2 cells (Xu et al., 2023).

These basic studies demonstrate that GLP-1RAs offer significant protection against obesity-related kidney disease, in addition to their benefits in improving DKD. Exenatide is even more effective than simvastatin in treating obesity-induced tubular epithelial cell lipotoxicity (Wang et al., 2021). The fact that GLP-1RAs has also become a second-line therapy for CKD expands its clinical benefits range besides weight loss (Navaneethan et al., 2023). Moreover, it has been reported that liraglutide, either alone or in combination with rabeprazole, can protect against cisplatin-induced nephrotoxicity (Sharaf et al., 2023), highlighting the potential of GLP-1RAs for further validation in clinical trials investigating nephrotoxicity associated with antineoplastic drugs.

#### 3.2.3 DPP-4i

##### 3.2.3.1 Clinical trial

The outcomes of clinical trials assessing the impact of DPP-4i on renal outcomes remain controversial. Initial findings indicated the potential benefits of DPP-4i in ameliorating DKD. In a retrospective analysis of four clinical datasets concerning linagliptin, it was observed that treatment with linagliptin led to a significant reduction in UACR after 12–24 weeks (Groop et al., 2013). Other studies showed that linagliptin reduced the probability of first adverse kidney events (HR, 0.84 [95% CI, 0.72–0.97]; *p* = 0.02) and new-onset albuminuria (HR, 0.82 [95% CI, 0.69–0.98]; *p* = 0.03) (Cooper et al., 2015). In the GUARD study, gemigliptin improved microalbuminuria (decrease in UACR was −41.9 mg/g creatinine; *p* = 0.03) and macroalbuminuria (decrease in UACR was −528.9 mg/g creatinine; *p* < 0.001) in both the short-term 12-week observation and the 40-week extension study (Table 2) (Yoon et al., 2017; Han et al., 2018). Analysis of the SAVOR-TIMI 53 trial found that saxagliptin improved UACR in patients with renal insufficiency (the difference in UACR change was −19.3 mg/g; *p* = 0.033) (Mosenzon et al., 2017). In the CARMELINA trial, linagliptin had a significant advantage in reducing UACR in T2DM patients with or without nephrotic range proteinuria (reduction of UACR ≥50%; HR, 1.15 [95% CI, 1.07 to 1.25] from baseline) (Wanner et al., 2021). In line with the CARMELINA trial, the EXAM trial showed that alogliptin may benefit patients with eGFR ≥60 mL/min/1.73 m^2^ (HR, 0.81 [95% CI, 0.65 to 0.99] for eGFR ≥60 mL/min/1.73 m^2^; HR, 1.2 [95% CI, 0.95 to 1.53] for eGFR <60 mL/min/1.73 m^2^) (Ferreira et al., 2020). However, there is contrary evidence to the above results. In the TECOS trial, sitagliptin did not significantly improve CKD progression, regardless of the baseline eGFR level (Cornel et al., 2016). The secondary analysis of CARMELINA also proved that linagliptin was not significantly different from placebo in improving renal outcomes (Perkovic et al., 2020). Postprandial glomerular hyperfiltration may be one of the renal risk factors in diabetic patients. Compared with glimepiride, linagliptin does not improve postprandial hemodynamics, and may even moderately induce postprandial glomerular hyperfiltration (Muskiet et al., 2020; Muskiet et al., 2022).

##### 3.2.3.2 Basic research

Inconsistent with clinical trials, preclinical data have unequivocally demonstrated the beneficial effects of DPP-4i in alleviating DKD. High glucose activates the C-reactive protein (CRP)/FcγRIIb (CD32b)/NF-κB pathway, which enriches DPP-4 and forms a dimer with CD32b to maintain its expression, thereby forming an inflammatory cycle and aggravating the injury. Linagliptin can block this cycle (Tang et al., 2021). Omarigliptin can improve high glucose-induced glomerular endothelial cell inflammation by activating the AMPK/mTOR pathway and negatively regulating the NLRP3 inflammasome (Figure 3B) (Li L. et al., 2021). Protein tyrosine phosphatase 1B (PTP1B) participates in the inflammatory response by negatively regulating the janus tyrosine kinase/signal transducer and activator of transcription (JAK/STAT) pathway. Sitagliptin reduces renal inflammation in streptozotocin-induced rats by inhibiting PTP1B (Table 3) (Al-Qabbaa et al., 2023). Linagliptin alleviate renal fibrosis in streptozotocin-induced mice by increasing the expression of microRNA 29. Upregulation of microRNA 29 directly inhibited the expression of fibrosis genes (Kanasaki et al., 2014).

DPP-4i may also alleviate renal dysfunction induced by AngII. The expression of Ang Ⅱ receptor 2 (AT2R), which can antagonize Ang Ⅱ receptor 1 (AT1R)-mediated inflammatory responses, was upregulated by linagliptin to alleviate Ang Ⅱ-induced renal fibrosis (Bai et al., 2020). Additionally, saxagliptin can mediate innate and adaptive immune inflammation, inhibit the activity of pro-inflammatory cells (CD8^+^T cells, neutrophils), and convert them into anti-inflammatory cells (M2 macrophages and Treg cells) to reduce Ang Ⅱ-induced hypertensive nephropathy (Nistala et al., 2021). In addition, saxagliptin can also activate multiple pathways, such as GLP-1/cAMP/VEGF, kidney injury molecule-1 (KIM-1)/STAT3/HIF-1α/VEGF/eNOS, to increase the expression of NO and repair damaged blood vessels caused by inflammation after renal ischemia/reperfusion (Kamel et al., 2019).

Additionally, DPP-4i play a significant role in improving antibiotic-induced nephrotoxicity and nephritis. Saxagliptin can reduce the expression of malondialdehyde and increase the expression of glutathione to regulate the disorder of renal inflammation and oxidative stress caused by gentamicin (Helal et al., 2018). Interestingly, linagliptin also accelerated glomerular crescentic degeneration in anti-glomerular basement membrane (GBM) nephritis (Mayer et al., 2021).

Basic research are still ongoing to explore the potential benefits and mechanisms of DPP-4i in improving DKD. Additionally, DPP-4i can improve hypertensive nephropathy through immune mechanisms independent of blood pressure reduction (Nistala et al., 2021), and promote the regression of crescents in anti-GBM nephritis, thereby providing a clinical translation point for their future use in immune system diseases.

## 4 Conclusion

Patients with T2DM often suffer from adverse cardiovascular and renal outcomes. Accumulating evidence suggest that SGLT2i and GLP-1RAs have cardiorenal protective effects including glucose-dependent and independent pathways. They not only protect against heart and kidney diseases through classical anti-inflammatory, anti-oxidative stress, and anti-fibrosis pathways but are also implicated in non-classical epigenetics, mitochondrial energy metabolism, and immune complement pathways. They have also demonstrated positive effects on immune diseases and cardiovascular and renal toxicity caused by antineoplastic drugs and antibiotics. Although basic research indicate the beneficial effects of DPP-4i, most clinical studies have only demonstrated their non-inferiority, underscoring the necessity for further exploration. Therefore, more direct and larger clinical trials (involving a larger proportion of CVD/CKD patients without diabetes) are needed to assess this drug.

By exploring the cardiorenal protective effects of drugs, we can identify common mechanisms that contribute to cardiorenal injury in various diseases. These findings will establish a theoretical and experimental basis for developing novel clinical drugs. Additionally, a drug that can effectively treat both heart and kidney diseases has significant practical implications, including reducing the medication burden on patients, lowering adverse reactions, enhancing patient compliance, and alleviating financial strain and so on. Therefore, further research should investigate new mechanistic pathways to explore the effectiveness of second-generation anti-glucose drugs.
